# Nuclear envelope: a new frontier in plant mechanosensing?

**DOI:** 10.1007/s12551-017-0302-6

**Published:** 2017-08-12

**Authors:** Kateryna Fal, Atef Asnacios, Marie-Edith Chabouté, Olivier Hamant

**Affiliations:** 1 Laboratoire Reproduction et Développement des Plantes, Université de Lyon, ENS de Lyon, UCB Lyon 1, CNRS, INRA, 69342 Lyon, France; 2 Laboratoire Matières et Systèmes Complexes, Université Paris-Diderot and CNRS, UMR 7057, Sorbonne Paris Cité, Paris, France; 3 Institut de Biologie Moléculaire des Plantes, CNRS, Université de Strasbourg, 67000 Strasbourg, France

**Keywords:** Nuclear envelope, Lamina, LINC complex, Cytoskeleton, Chromatin, Mechanical force, Microrheology, Plants

## Abstract

In animals, it is now well established that forces applied at the cell surface are propagated through the cytoskeleton to the nucleus, leading to deformations of the nuclear structure and, potentially, to modification of gene expression. Consistently, altered nuclear mechanics has been related to many genetic disorders, such as muscular dystrophy, cardiomyopathy and progeria. In plants, the integration of mechanical signals in cell and developmental biology has also made great progress. Yet, while the link between cell wall stresses and cytoskeleton is consolidated, such cortical mechanical cues have not been integrated with the nucleoskeleton. Here, we propose to take inspiration from studies on animal nuclei to identify relevant methods amenable to probing nucleus mechanics and deformation in plant cells, with a focus on microrheology. To identify potential molecular targets, we also compare the players at the nuclear envelope, namely lamina and LINC complex, in both plant and animal nuclei. Understanding how mechanical signals are transduced to the nucleus across kingdoms will likely have essential implications in development (e.g. how mechanical cues add robustness to gene expression patterns), in the nucleoskeleton–cytoskeleton nexus (e.g. how stress is propagated in turgid/walled cells), as well as in transcriptional control, chromatin biology and epigenetics.

## Introduction

Plants, like animals, respond to mechanical stimuli. This is probably most obvious when looking at a section of a tree branch: its anatomical asymmetry reveals the existence of so-called “reaction wood”, the product of an active mechanical reinforcement that matches the asymmetric load caused by gravity. In recent years, plant mechanosensing research is turning more and more towards cell and molecular aspects, from cytoskeleton behaviour to the regulation of gene expression following mechanical perturbations (Braam 2005; Hamant 2013; Monshausen and Haswell 2013; Coutand et al. 2009; Geitmann 2010). Although plants exhibit specific cell features, like a stiff cell wall (in the MPa range) and high hydrostatic pressure (turgor pressure, also in the MPa range), both kingdoms display a number of comparable responses to mechanical cues.

Animal cells respond to their mechanical environment, notably through interactions with their extracellular matrix (see e.g. Vogel and Sheetz 2006, 2009; Discher et al. 2005, 2009). Plant and animal extracellular matrices are structurally and chemically very different. From the signalling point of view, the quasi absence of true integrins in plant genomes (for an exception, see Knepper et al. 2011) needs to be put in context against the high number of receptor-like kinase in plants. Several of these proteins can interact with wall components (e.g. the WAK receptor with the backbone of pectins; Anderson et al. 2001; Wolf et al. 2012), a bit like integrin with fibronectin, arguably. Interestingly, one such receptor-like kinase, FERONIA, contributes to mechanoperception in *Arabidopsis* roots (Shih et al. 2014).

At the plasma membrane, a role of tension in cell polarity has been shown in both kingdoms, notably through the inhibitory role of membrane tension on endocytosis, that can trap transporters (e.g. Heisler et al. 2010; Nakayama et al. 2012) or receptors (e.g. Pouille et al. 2009) in polar domains (for a comparative review between plants and animals, see Asnacios and Hamant 2012). Similarly, in both kingdoms, membrane tension should lead to membrane thinning, which, in turn, changes the conformation of mechanosensitive channels, leading to their opening (Haswell et al. 2011).

Inside the cell, the cortical cytoskeleton is a focus of mechanotransduction research in both plants and animals. However, one must highlight here that most animal cells exhibit an actomyosin-rich cortex, consistent with their contractility, whereas plant cells have a microtubule-rich cortex: cortical microtubules (CMTs) guide the cellulose synthase complex at the plasma membrane, thus channelling the production of cellulose microfibrils in the wall (Green 1962; Paredez et al. 2006). Both actomyosin and microtubules respond to mechanical cues. In animal cells for instance, myosin is preferentially recruited on tensed membrane, providing a positive feedback loop for cell contraction, amenable to generating tissue folding (Lecuit and Lenne 2007; Sherrard et al. 2010). In plants, cortical microtubules are oriented in the direction of maximal tension, thereby controlling the deposition of stiff cellulose microfibrils, through CMT–cellulose synthase complex guidance, in the wall (Green and King 1966; Williamson 1990; Hejnowicz et al. 2000). Interestingly, this mechanical feedback has also been proposed to enhance tissue folding in plant tissues, through the local channelling of growth direction (Hamant et al. 2008; Uyttewaal et al. 2012).

Mechanical stimuli at the extracellular matrix, membrane and cytoskeleton may be transmitted to the cell nucleus, notably because the nuclear envelope is physically interacting with the cytoskeleton (Ingber 2003; Wang et al. 2009; Dahl et al. 2010; Fedorchak et al. 2014). Yet, despite the established impact of mechanical forces on gene expression in all kingdoms, the nexus between mechanical stress and nucleus remains largely unexplored in plants. Nonetheless, some recent results may point in that direction, albeit quite indirectly. The chromatin modifying enzyme SDG8 is required for the control of gene expression in response to touch (Cazzonelli et al. 2014). More recently, in a screen for touch-insensitive mutants, the transcriptional regulator VIP3, a member of the Paf1 complex, was identified (Jensen et al. 2017), suggesting that nuclear factors might have a stronger role in mechanotransduction than anticipated. Here, we propose to investigate the possible contribution of the nuclear envelope in that framework. To do so, we discuss biophysical methods to probe plant nuclei and the coupling between cytoskeleton and nucleoskeleton; we also compare the putative pathways and molecular targets involved in nuclear mechanotransduction in plant and animal cells (Table 1).

**Table 1:** Molecular players involved in mechanotransduction at the nucleus

Name of complex	In animals	In *Arabidopsis*	Function (shown in animals^a^ or plants^b^)	References
LINC complex	SUN (Sad1–UNC-84) UNC-84 (*C. elegans*) SUN3-5 (human)	AtSUN1, AtSUN2, AtSUN3, AtSUN4, AtSUN5	Nuclear shaping^ab^, interacts with lamins in the nucleoplasm^a^, stabilises the nuclear envelope against cytoplasmic forces^a^, facilitates nuclear positioning and movement^a^, interacts (SUN1) with the plamina candidate, CRWN1^b^	Apel et al. (2000), Hagan and Yanagida (1995), McGee et al. (2006), Murphy et al. (2010), Göb et al. (2010), Graumann et al. (2010, 2014), Chi et al. (2012)
	KASH (Klarsicht, ANC-1 and Syne homology) proteins - nesprins UNC-83 (*C. elegans*)	AtSINE1, AtSINE2, AtSINE3, AtSINE4, AtTIK, AtWIP1, AtWIP2, AtWIP3	Connects the inner nuclear envelope to the actin cytoskeleton^ab^ (AtSINE1, AtWIP), involved in nuclear movement^ab^ (AtWIP) and positioning^ab^ (AtSINE1)	Zhou et al. (2012, 2015), Graumann et al. (2014), McGee et al. (2006), Apel et al. (2000), Mosley-Bishop et al. (1999), Mislow et al. (2002), Zhen et al. (2002), Padmakumar et al. (2004, 2005), Crisp et al. (2006), Hodzic et al. (2004), Starr and Fridolfsson (2010), Tamura et al. (2013), Zhou et al. (2014)
Lamina and lamine-like, associated proteins	A-type lamins A, C, AΔ10 and C2, B-type lamins B1 and B2/B3	AtCRWN1, AtCRWN4, AtKAKU4, AtNEAP1, AtNEAP2, AtNEAP3	Structural network at the inner nuclear membrane^ab^, contributes to maintaining the nuclear shape^ab^, size^ab^ and stiffness (lamins A, C)^a^	Stewart and Burke (1987), Höger et al. (1988), Lin and Worman (1995), Liu et al. (2000), Lammerding et al. (2006), Biamonti et al. (1992), Dittmer and Misteli (2011), Gruenbaum and Foisner (2015), Zwerger and Medalia (2013), Dittmer et al. (2007), Pawar et al. (2016), Goto et al. (2014)
Regulator of γ-tubulin complex	MOZART1 and MZT1	AtGIP1, AtGIP2	Associated with GCP3^ab^, regulates the recruitment of γ-tubulin complex at the MTOC^a^ and the stability of the microtubule network^ab^, regulates microtubule nucleation^a^, located on both sides of the nuclear envelope^b^, involved in nuclear shaping^b^ and centromere organisation^b^	Hutchins et al. (2010), Dhani et al. (2013), Janski et al. (2008, 2012), Nakamura et al. (2012), Masuda and Toda (2016), Lin et al. (2016), Batzenschlager et al. (2013, 2015), Cota et al. (2017)

## The molecular players of mechanotransduction at the nuclear envelope in animal cells

**Fig. 1:**
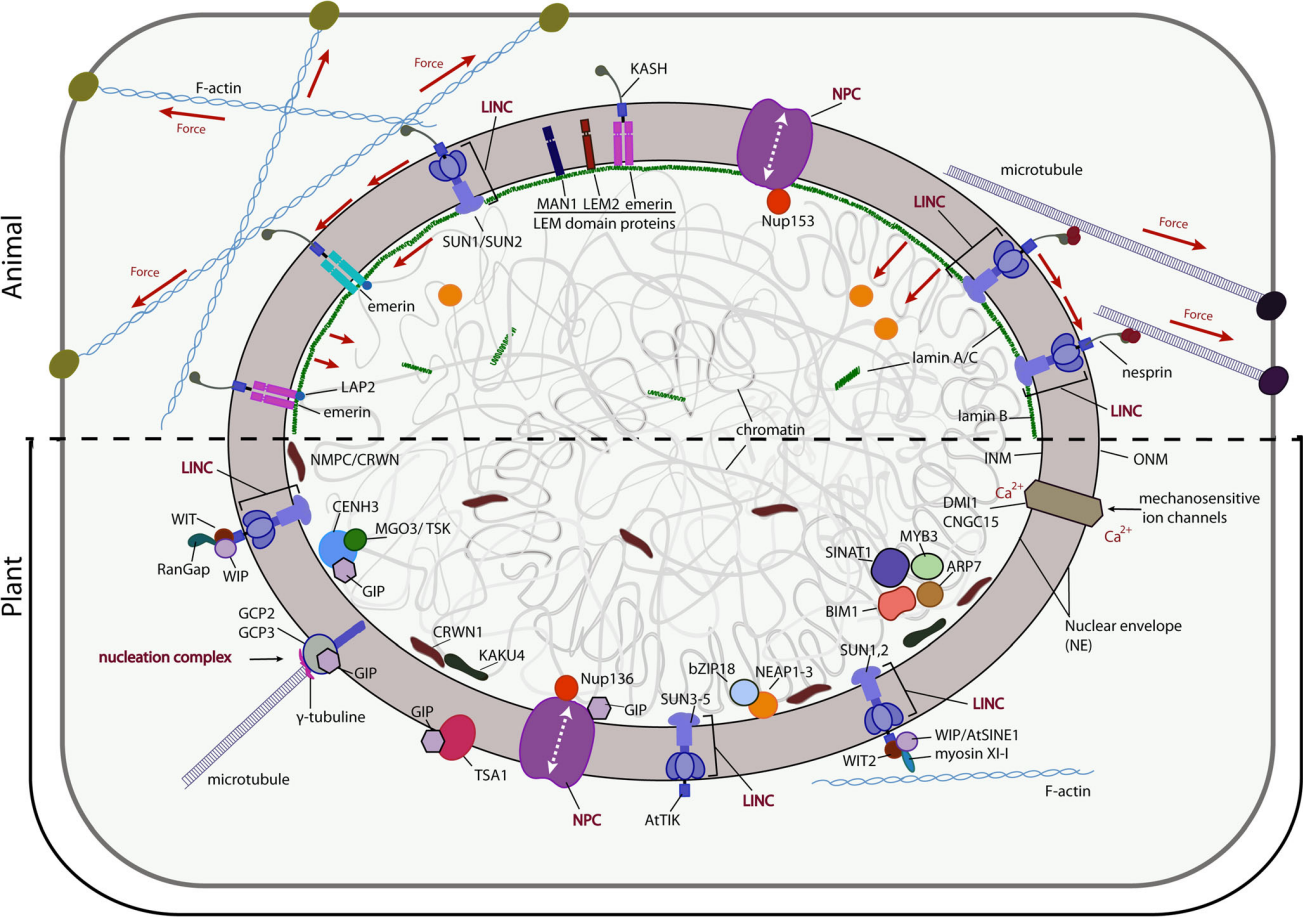
Mechanosensing at the nuclear envelope in animals and candidates in plants. The upper part of the figure illustrates the elements involved in nucleus mechanosensing in animals; the lower part is dedicated to the putative components of mechanosensing at the plant nucleus. The nuclear pore complexes, anchored to nuclear envelope (NE) ensure the selective transport of molecules through the inner and outer nuclear membranes (INM and ONM, respectively). At the nuclear pore complex, nucleoporins Nup153 (animals) and Nup136 (plants) have been identified. LINC complexes are composed of KASH domain nesprins and SUN domain-containing proteins in animals. In plants, LINC complexes are represented by SUN proteins (animal homologues) and KASH proteins (e.g. WIP, WIT), with no structural homology to their functional equivalents in animals. The LINC complex couples the cytoskeleton to the nucleoskeleton. The inner nuclear membrane of animal nuclei harbors the LEM domain family proteins (LEM2, MAN1, emerin) that interact with lamins at the periphery of the nucleus. At the inner membrane of the plant nucleus, the NEAP1–3 proteins interact with bZIP18 and chromatin. The plant lamin-like nuclear matrix components (NMCPs), also called crowded nuclei (CRWN), have several interactors (KAKU4, ARP7, BIM1, MYB3 and SINAT1). Plant ion channel complex comprising DMI1-CNGC15 is localised on both sides of the nuclear envelope. GIP proteins, present on both sides of the nuclear envelope, function as a component of microtubule nucleation complexes (at the outer nuclear membrane), they are associated with the nuclear pore complex and TSA1, and colocalise with centromeres and epigenetic regulator MGO3/TSK

The structure of the nuclear envelope is stabilised by a network of integral proteins, anchored to the inner nuclear membrane (Zwerger and Medalia 2013; Gruenbaum and Foisner 2015). Among those, numerous proteins and protein complexes ensure continuous selective transport of molecules between the cytoplasm and caryoplasm (Schirmer et al. 2003; Korfali et al. 2012). Nuclear pore complexes prevent the free diffusion of macromolecules (radius ≥ 2.5 nm, corresponding to a protein of ca. 35–40 kDa in mass). They are composed of multiple constituent proteins (nucleoporins or Nups), containing the phenylalanine-glycine (FG) repeats and anchored to a membrane of the nuclear envelope (Field et al. 2014; Obado et al. 2016; Mohr et al. 2009). The transport of large macromolecules through the nuclear envelope is supported by nuclear transport proteins (NTRs, importins/exportins) that bind the target cargo molecules and assist their passage through the FG repeat-rich nuclear pore complex core. One of the most notable players in the asymmetric directional transport, Ran GTPase, has its GTP-bound form in the nucleus opposing the GDP-bound one in the cytoplasm (Schmidt and Görlich 2016). Disruption of nuclear pore complexes following an oxidative stress had been shown to result in nuclear aggregation of cytosolic proteins, a phenotype associated with neurodegenerative disease (D’Angelo et al. 2009). Although nuclear pore complexes may have an indirect role in shaping nuclei and mechanosensing, here, we will focus on the two main structural components of the nuclear envelope, namely the LINC complex and the lamina (Fig. 1).

The LINC complex consists of SUN (homologous to Sad1p and UNc-84) and Klarsicht/ANC-1/Syne-1 homology (KASH) proteins, which span the nuclear envelope and connect the nucleoskeleton with the cytoskeleton.

Mammalian nesprin belong to a large family of actin-binding proteins, encoded by nesprin1 and 2 genes. Containing the c-terminal KASH domain, they are homologous to *Drosophila melanogaster* protein *Klarsicht* and are located at the outer nuclear membrane (Mosley-Bishop et al. 1999; Apel et al. 2000; Mislow et al. 2002; Zhen et al. 2002; Padmakumar et al. 2004, 2005).

Several SUN homology domain proteins have been identified in mammalian cells (Hagan and Yanagida 1995). SUN1 and SUN2 have a considerable degree of functional redundancy. They localise to the inner nuclear membrane and interact with lamins (see below) by KASH domain proteins, connecting the inner nuclear envelope to the actin cytoskeleton (Crisp et al. 2006; Padmakumar et al. 2004, 2005; Hodzic et al. 2004; Starr and Fridolfsson 2010). The localisation of SUN1 near nuclear pore complexes at the inner nuclear membrane may reflect a role of SUN1 for the recruitment of pre-lamins for nuclear lamina assembly at NPCs (Liu et al. 2007). On the contrary, SUN2 are located in NPC-free regions and the nesprin2 Giant (NUANCE) and SUN2 have been shown to colocalise with actin during the nuclear movement in polarising fibroblasts (Lombardi et al. 2011; Arsenovic et al. 2016; Khatau et al. 2012; Lüke et al. 2008; Thorpe and Lee 2017) and nesprin3 was shown to be required for actin remodelling and cell polarisation in response to shear stress (Morgan et al. 2011). The controlled migration of nuclei in the *C. elegans* P cells (3–4 mm diameter moving over 150 nm between the body wall muscle and the worm’s cuticle) is dependent on interactions between canonical SUN and KASH proteins, UNC-84 and UNC-83 (microtubule recruitment) or ANC-1 (actin recruitment) (Stewart-Hutchinson et al. 2008). Recently, human muscle cell precursors were shown to require nesprin1 to sense the stiffness of the extracellular matrix, highlighting the central role of the LINC complex and force transmission through the nuclear membrane for the cell to respond and adapt to its mechanical environment (Schwartz et al. 2017).

The LINC complex is sensitive to low stress magnitude (Chambliss et al. 2013). In particular, low shear stresses may only activate the actin cap-based physical pathway (small subset of actin fibres connected to the nuclear envelope through the LINC complex, NUANCE and nesprin3 that form the perinuclear actin cap), while high shear stresses would engage both this LINC/actin cap-based physical pathway as well as previously established biochemical pathways, such as the integrin activation through the c-Src and phosphoinositide 3-kinase cascade (Shyy and Chien 2002; Tzima et al. 2005), NADPH oxidase inactivation (Godbole et al. 2008) and prostaglandins induction through the cyclooxygenase-2 (Di Francesco et al. 2009) (also reviewed in Lu and Kassab 2011).

Inside the nucleus, the lamina is composed of helix-rich fibrillar lamin proteins that form a structural network near the inner nuclear membrane (Goldman et al. 2002; Gruenbaum and Foisner 2015; Zwerger and Medalia 2013). The lamina maintains the nuclear shape and size, and is indirectly related to the cytosolic cytoskeleton via the LINC complex (Crisp et al. 2006). Lamins assist the recruitment of the LEM (LAP2, emerin and MAN1) family to the nuclear envelope and take part in nuclear pore complex stabilisation (Gesson et al. 2016; Margalit et al. 2005; Shaklai et al. 2007; Shimi et al. 2015; Xie et al. 2016). They are also involved in chromatin organisation and gene regulation (through heterochromatin-associated proteins and because certain transcription factors can be harboured at the nuclear envelope), as well as nuclear mechanical stability (Schirmer et al. 2003; Shimi et al. 2015; Korfali et al. 2012; Gruenbaum and Foisner 2015; Xie et al. 2016; Paddy et al. 1990; Solovei et al. 2013; Margalit et al. 2005; Wilson and Foisner 2010).

In mammals, two types of lamins have been identified. Lamins A, C, AΔ10 and C2 belong to the A-type and are the products of the alternative splicing of a single gene, *LMNA* (Nakajima and Abe 1995; Peter et al. 1989, reviewed in Dittmer and Misteli 2011). They are expressed in differentiated and developmentally regulated cells (Furukawa et al. 1994; Lin and Worman 1993; Liu et al. 2000). Another group, the B-type lamins, comprises B1 and B2/B3 proteins, are encoded by two independent genes (*LMNB1* and *LMNB2*) and are constitutively expressed in all cell types (Peter et al. 1989; Stewart and Burke 1987; Höger et al. 1988; Lin and Worman 1995; Liu et al. 2000; Biamonti et al. 1992, reviewed in Dittmer and Misteli 2011). In murine dermal fibroblasts, lamin A/C is localised throughout the nucleus, associates with the chromatin-binding protein lamina-associated polypeptide (LAP) 2α and interacts with euchromatin (Gesson et al. 2016). In contrast, lamin B1 has been mainly detected at the nuclear periphery and was only found to be associated with heterochromatin. In LAP2α-deficient cells, loss of lamin A/C at heterochromatic regions is correlated with increased gene expression, suggesting a role of lamins A/C in euchromatin regulation (Gesson et al. 2016; Shaklai et al. 2007).

The amount of lamin A positively correlates with nuclear and tissue stiffness, and deficiency in lamins A/C has been associated with distorted and fragile nuclei (Liu et al. 2000; Vigouroux et al. 2001; Swift et al. 2013); therefore, it has been suggested that lamins A/C play a role in the nucleus response to mechanical strain. The depletion of lamins A/C results in significant decrease of nuclear stiffness, highlighting their primary role in nuclear structure, while also indicating a contribution of other nuclear components to the remaining stiffness (Dahl et al. 2005; Pajerowski et al. 2007; Lammerding et al. 2006). The loss of LINC complex or the actin bundles does not rescue nuclear lamina defects; however, it leads to a decrease of size and quantity of chromatin hernias (i.e. chromatin exiting the nucleus upon nuclear envelope rupture) (Hatch and Hetzer 2016). In contrast, the nucleus rupture in cells treated with actin-depolymerising drugs could be rescued by mechanically constraining the nucleus (Hatch and Hetzer 2016). Although this is debated, the rupture of nuclear envelope with defects in lamina organisation could be caused by an increase in intranuclear pressure from actin-based nucleus confinement (Furusawa et al. 2015; Schreiner et al. 2015; Hatch and Hetzer 2016). Interestingly, recent experiments demonstrated that the nucleus exhibits two deformation regimes in response to mechanical strain. The main component that contributes to maintenance of nuclear shape at small deformations was identified to be the chromatin itself, while lamins A/C play a role in the stiffening of nuclei when subjected to larger deformations (Stephens et al. 2017).

## Microrheometry to analyse nucleus mechanics and the coupling between cytoskeleton and nucleoskeleton

**Fig. 2:**
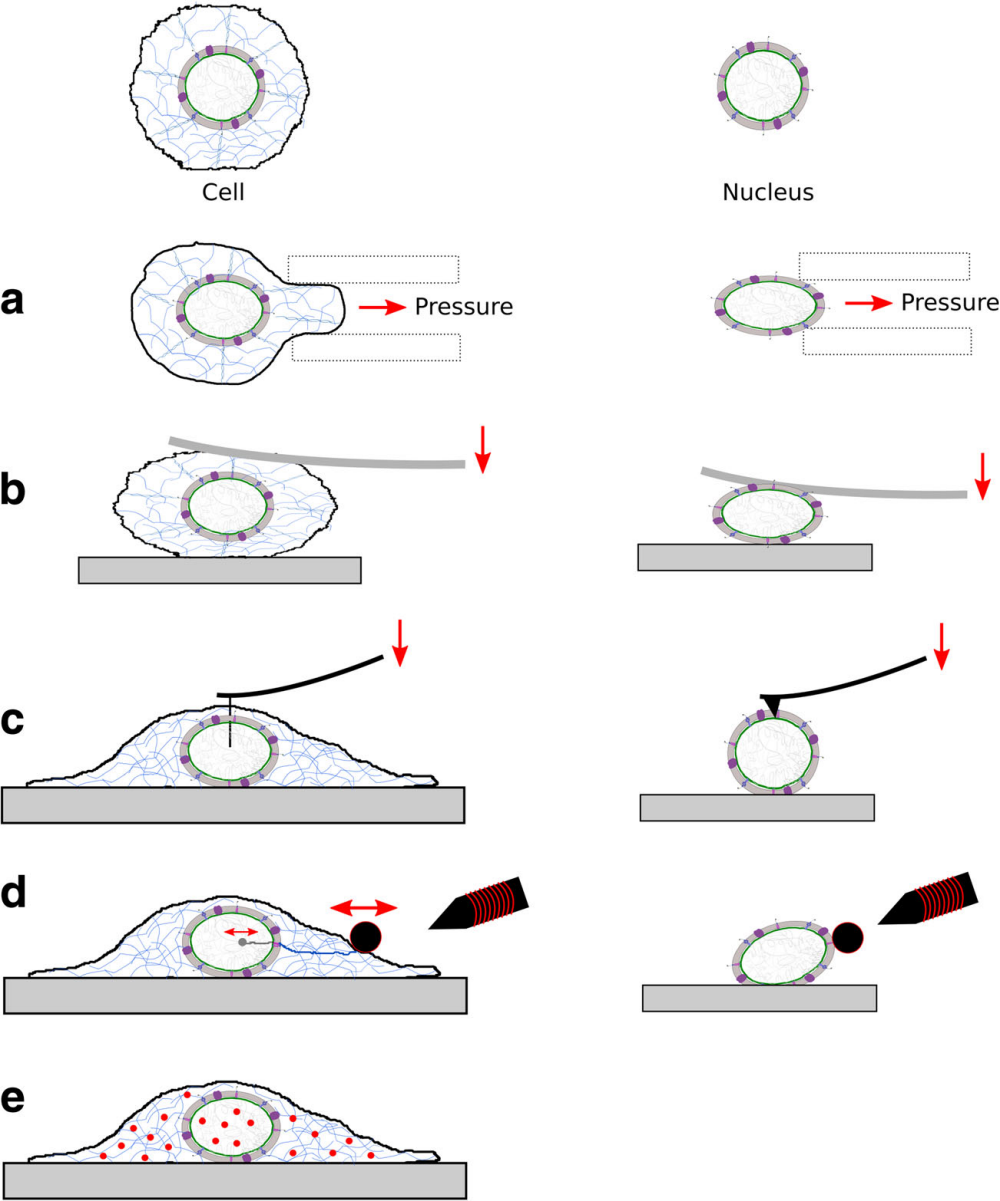
Mechanical measurements on whole cells (*left*) and on isolated nuclei (*right*). **a** Cell/nucleus deformed by micropipette aspiration. **b**, **c** Techniques based on cantilever (spring of calibrated stiffness) deflection. **b** Compression between microplates, global deformation. Micropipettes and parallel microplates allow direct comparison between cell and nucleus mechanics. **c** Local probing of the nucleus. Left: in situ characterisation of the nucleus mechanical properties using a custom-made sharp atomic force microscope tip to penetrate cell and nucleus membranes. Right: regular AFM tip used to probe the nucleus surface. **d** Magnetic bead-based microrheometry. Left: a twisting magnetic field applies oscillations on a bead bound to the cell surface and oscillations are transmitted to nuclear components through the cytoskeleton, the LINC complex and the lamina. Right: successive current pulses lead to repeated magnetic traction forces applied on a bead bound to the nucleus surface, causing nucleus stiffening (mechanosensing). **e** Passive microrheology based on nano-particle tracking. Comparison between particle movements in the cytoplasm and the nucleus helps to characterise the link between the cytoskeleton and nucleoskeleton

Beyond the identification of the molecular players at the nuclear envelope, their formal integration with mechanical cues has been possible thanks to the development of micromechanical methods and techniques. As shown above, a key landmark in this endeavour was the identification of a central role of the LINC complex in mechanotransduction from cell surface to chromatin (Dahl and Kalinowski 2011). Before investigating this question in plant nuclei, we review here some of the methods that have been used to analyse the nuclear mechanics of animal cells, focusing on the most direct method, micrometry (Box 1, Fig. 2).

**Box 1. Rheometry**

**Table:** 

Rheometry, from the Greek word “rheos” (flow), is the discipline dedicated to the quantitative characterisation of the rheological properties of materials, i.e. the way they deform and flow when submitted to external forces. Rheometry techniques can essentially be divided into two classes: active and passive rheometry. In active rheometry, one applies a stress (force per unit area, dimension of a pressure), either constant (static) or variable (dynamic rheometry), and measures the induced sample strain (dimensionless deformation, i.e. as a percentage of the initial sample size). Basically, the relationship between stress and strain defines the mechanical behaviour of the sample, which is quantified by a modulus (elastic and/or viscous, with dimension of a pressure). Most studies on nuclear mechanics were carried out with active microrheometry techniques. In passive microrheometry, the spontaneous movement of nanoparticles inside the cytoplasm and/or the nucleus (either injected synthetic ones or components of the nuclear material) is tracked and viscoelastic moduli are calculated from the mean square displacement (MSD, a measure of the mean distance travelled by a particle after a given time).	

Among micromechanical techniques, micropipette aspiration is probably the one that was used most often for nuclear mechanics characterisation. Rheometric measurements with micropipettes were first done on single cells (Hochmuth 2000), but were rapidly extended to isolated nuclei since micropipettes constitute an “all-in-one” rheometer and micromanipulation tool (Guilak et al. 2000): by applying a pressure drop on a micrometric pipette, one can easily aspirate a single nucleus and monitor its elongation in the glass tube (e.g. with fluorescence microscopy) and observe the relevant to concomitant deformation of a given element of the nucleoskeleton. In particular, micropipette aspiration was successfully used to define the role of lamins in nuclear mechanics (Dahl et al. 2004, 2005), and their implication in differentiation (Pajerowski et al. 2007; Shin et al. 2013), their adaptation to the rigidity of the extracellular matrix and tissues (Swift et al. 2013; Buxboim et al. 2014), as well as their involvement in major diseases (e.g. Dahl et al. 2006).

Beyond the focus on lamins from the mechanics of isolated nuclei, micropipettes were also used to deform nuclei inside living cells, in order to determine the proteins involved in the mechanical coupling between the cytoskeleton and the nucleoskeleton. For instance, nuclei lacking emerin (linker protein from the inner nuclear membrane) were shown to display altered elasticity (Rowat et al. 2006). More recently, the ability of nuclei to recover from micropipette-imposed deformations was shown to depend on intermediate filaments, SUN proteins and lamins, but neither on microtubules nor actin filaments (Neelam et al. 2015).

In parallel to micropipette measurements, different cantilever-based techniques were used to compress (global scale deformation: parallel microplates, compressive cell device) or to indent (local deformation: atomic force microscope) cells or isolated nuclei (Bao and Suresh 2003; Thoumine et al. 1999; Broers et al. 2004; Schäpe et al. 2009). In these techniques, the cantilever (basically a spring of calibrated stiffness) is deflected to apply a well-defined stress on the sample, and the strain is determined through image analysis, from cantilever deflection or/and displacement, depending on the particular technique and protocol. Parallel microplates measurements were among the first to show mechanical continuity between cytoskeleton and nucleoskeleton, and to quantitatively compare their elastic moduli, the nucleus being about ten times stiffer than the cytosol (Thoumine et al. 1999; Caille et al. 2002). As mentioned above, the observation that cells lacking lamin A exhibit lowered stiffness and bursting force than wild-type counterparts was notably shown using compressive cell device measurements on mouse embryonic fibroblasts, indicating that the nucleus contributes to the overall cell resistance to deformation, in line with clinical phenotypes observed in muscles dystrophies due to mutation in the lamin A/C gene (laminopathies) (Broers et al. 2004).

When used to measure the local rheological properties of isolated nuclei, atomic force microscopy led to results comparable to those retrieved from global probing with micropipettes, with a prominent role of lamins in stiffening the nuclear envelope and nucleus (Dahl et al. 2005; Schäpe et al. 2009). Interestingly, by probing nuclei from *Drosophila* embryos, it was shown that the inner nuclear membrane protein Kuk stiffens the nuclear envelope and controls its shape through coupling to polymerising microtubule bundles (Hampoelz et al. 2011). Beyond these atomic force microscopy measurements on isolated nuclei, a custom-made sharp-needle atomic force microscopy probe has been recently introduced to measure nucleus modulus in situ, by penetrating the cell and nuclear membranes with minimal injury. The authors showed that cell-embedded nuclei are stiffer than isolated ones, probably due to strain stiffening, i.e. tension transferred to the nucleus from the cytoskeleton (Liu et al. 2014).

Among local rheometric measurements, magnetic twisting cytometry is certainly one of the most popular in the field of cell rheology (Fabry et al. 2001). A ferromagnetic bead bound to cell-surface integrins is twisted thanks to a rotating magnetic field, leading to bead displacement and cyclic deformation of the cell cytoskeleton, which is anchored to surface adhesion complexes. However, in the context of nuclear mechanics and mechanosensing, magnetic twisting cytometry was mainly used as a means to test the hypothesis of a directed force transmission from cell-surface receptors to the nucleus through the tensed cytoskeletal polymer network. Indeed, while applying small cyclic bead displacements at the cell surface (in the range of ~0.4 μm), stress was found to propagate inside the nucleus, the nucleoli being deformed upon external mechanical stimulation (Maniotis et al. 1997; Hu et al. 2005). Protein complexes from the Cajal body could also undergo cyclic stretch until complete dissociation (Poh et al. 2012).

A slightly different version of magnetic bead-based microrheometers, called magnetic tweezers, uses controlled electric currents to create variable magnetic field gradients to pull on magnetic beads bound to the cell surface (Bausch et al. 1998). Combining magnetic tweezers with cell culture on stretchable membranes, mouse embryonic fibroblasts lacking lamin A were shown to have softer nuclei, as well as overall decreased cell stiffness (Lammerding et al. 2004), in line with the results observed with compression experiments (Broers et al. 2004), underlining, once again, the nuclear contribution to whole-cell mechanics and its possible role in muscular tissue weakness in laminopathies. More recently, magnetic tweezer measurements were directly carried out on isolated nuclei (Guilluy et al. 2014) to reveal a nucleus-specific mechanotransduction pathway related to the LINC complex. Indeed, beads bound to nesprin1 and subjected to repeated magnetic pulses led to progressive stiffening of isolated nuclei. Moreover, this stiffening was independent of nuclear actin and chromatin, but required intact lamins and emrin, the latter being phosphorylated in response to force (Guilluy et al. 2014).

Passive microrheometry, based on the analysis of the movement of nanoparticles injected in the cytoplasm and/or the nucleus, was extensively used to investigate nuclear structure and mechanics (Tseng et al. 2004), and the specific roles of lamin A/C (Lee et al. 2007) and LINC complex (Hale et al. 2008) in the whole-cell mechanics, in particular adhesion, polarisation and migration. These intracellular nanoscale measurements confirmed the mechanical continuity of the cell structure from cytoskeleton to nucleoskeleton, and the central role of the LINC complex in mechanotransduction and global cell coordination. Of note, internal active microrheometry was also made possible by the injection of magnetic nanorods in the nuclei of adherent cells and their manipulation at a distance with rotating magnetic fields (Celedon et al. 2011).

While microrheology of the cell/nucleus and mechanotransduction were extensively studied in the past years in animal cells, no such effort has been invested for walled cells, in particular in plants. This is quite surprising since plants are obviously mechanosensitive. It has been even shown, at the cell scale, that forces applied at the surface of tobacco cells could induce migration of the nucleus (Qu and Sun 2007). This could be due to the fact that some of the micromechanical methods reviewed here would be inefficient (too weak) to investigate plant cell structures that are mechanically shielded by the huge turgor pressure and cell wall. However, one could argue that measurements could be carried out on wall-less plant protoplasts as well as on isolated nuclei. In that respect, comparison between the mechanics of animal and wall-less plant cells is instructive (Durand-Smet et al. 2014). It will also be of interest to mechanically characterise isolated plant nuclei, as well as their interaction with cytoskeleton polymers in vitro (Stoppin et al. 1994). Such studies will help define differences and putative conserved core mechanosensing mechanisms between plants and animals (Asnacios and Hamant 2012). A first step in this endeavour might be the identification of some key players in plant nuclei (Fig. 1).

## Plant homologues in nuclear envelopes

As in animals, plant nuclei display nuclear pore complexes; most of the nucleoporins are homologous to the vertebrate ones and contribute to the nucleocytoplasmic transport. Note that Nup136, which is unique to plants, dynamically interacts with the nuclear pore complex and may be considered as the functional homologue of the human Nup153 (Tamura et al. 2010).

Similarly, LINC complexes involving SUN–KASH proteins exist in plants, bridging the cytoskeleton to nucleoskeleton at the nuclear envelope. The SUN-interacting KASH proteins, located at the outer nuclear membrane, were mainly identified in *Arabidopsis*. They display a limited conservation with known opisthokont KASH proteins, except for AtTIK, which harbours a more classical KASH tail (Graumann et al. 2014). Specific SUN–KASH bridges were shown between AtSUN1, 2 proteins and AtWIP1-3, as well as between the mid-SUN AtSUN3-5 and AtTIK (Zhou et al. 2012; Graumann et al. 2014). Both AtWIPs and AtSINE1 are indirectly associated with actin filaments, notably through the physical interactions between ATWIT2 and the plant-specific Myosin XI-i (Tamura et al. 2013).

Although plant cells are devoid of a centrosome, the nuclear envelope constitutes a site of microtubule nucleation (Stoppin et al. 1994). γ-Tubulin complex proteins were identified in plants, including GCP2 and GCP3 proteins, which have nuclear targeting domains (Seltzer et al. 2007). GIPs (GCP3 interacting proteins) were first identified in plants as novel regulators of γ-tubulin complexes (Janski et al. 2008, 2012). Two GIP proteins are present in almost all plant genomes, whereas the GIP homologues in animals and *Schizosaccharomyces pombe*, called MOZART1 and MZT1, respectively, are single genes (Hutchins et al. 2010; Dhani et al. 2013). Contrary to the nuclear envelope functional components, GIPs are dynamic proteins found on both sides of the nuclear envelope, as well as at the nuclear pores (Batzenschlager et al. 2013). They interact with TSA1, which is located at the nuclear envelope and most probably the endoplasmic reticulum in interphase cells (Suzuki et al. 2005). Near the inner nuclear membrane, GIPs are located close to chromocentres and colocalise with centromeres (Batzenschlager et al. 2015). Because of the role of GIP in centromeric cohesion and in CENH3 loading, GIPs also contribute to centromere functions (Batzenschlager et al. 2013; Chabouté and Berr 2016). Therefore, the GIPs seem to bridge nuclear regulation and cytoplasmic microtubules at the nuclear envelope (Fig. 1).

At the inner nuclear membrane, *Arabidopsis* and maize SUN domain proteins exhibit the conserved features of their eukaryotes counterparts, with the existence of SUN1, SUN2 and mid-SUN homologues 3 to 5 (Graumann et al. 2010, 2014; Murphy et al. 2010). Whereas SUN1 and SUN2 are exclusively located at the nuclear envelope (Graumann et al. 2010), SUN3 and SUN4 share localisation between endoplasmic reticulum and nuclear envelope; SUN5 localisation has not yet been analysed (Graumann et al. 2014). The plant-specific NEAP1–3 proteins were also identified at the inner nuclear membrane in *Arabidopsis* and NEAP1 may be connected with chromatin through its interaction with a putative transcription factor (bZIP18, Pawar et al. 2016).

Near the inner nuclear membrane, a fibrous meshwork similar to the animal lamina was observed by field emission scanning electron microscopy (FE-SEM) in tobacco BY2 cells and was called “plamina” (Fiserova et al. 2009; Ciska and Moreno Díaz de la Espina 2014). While no homologues of lamins have been identified in plant genomes, functional candidates of the nuclear matrix beneath the nuclear envelope were characterised, such as the NMPC1 protein in carrot (Masuda et al. 1997), CRWN1, 4 (Dittmer et al. 2007) and KAKU4 (Goto et al. 2014) in *Arabidopsis*.

## Mechanosensing through the nuclear envelope: candidates in plants

Since structural changes of nuclear envelope proteins and chromatin are important features in mechanotransduction in animals and yeast (Dahl et al. 2008; Fedorchak et al. 2014), the following features will be considered as potential candidates in plant nuclear mechanosensing: nuclear deformability, link to nuclear envelope and chromatin remodelling.

All the nuclear envelope, the actors cited above are involved in shaping nuclei. In differentiated *Arabidopsis* cells, most of the corresponding mutants display smaller and spherical nuclei when compared to large elongated nuclei in the wild type (for a review, see Tamura et al. 2015). Interestingly, the *gip1gip2* nucleus shape is affected in both differentiated and undifferentiated cells, with enlarged nuclei exhibiting shape distortions such as lobes and indentations (Batzenschlager et al. 2013, 2014). Moreover *gip* mutants are also impaired in nuclear pore complex distribution and architecture. Interestingly, while some proteins, such as GIP1–2, SUN1–2, SUN4–5 and WIP1–3, share functional redundancy in shaping nuclei (Zhou et al. 2012; Graumann et al. 2014; Batzenschlager et al. 2013), other nuclear envelope proteins, such as SUN3, CRWN1, CRWN4, KAKU4, MYOSIN XI-I or Nup136, may have non-redundant functions (Tamura et al. 2010, 2013; Wang et al. 2013; Goto et al. 2014). More specifically, KAKU4-dependent nuclear deformation can be uncoupled from CRWN1 or CRWN4 (Goto et al. 2014), as SUN-WIP-WIT2-MyosinXI-i-dependent nuclear deformation can be uncoupled from CRWN1 (Zhou et al. 2015). Furthermore, CRWN1 and CRWN4 may have additive effects (Wang et al. 2013) since nuclear shape defects are stronger in the double mutant than in single mutants. In addition, in either *wit1wit2* or *myosin XI-i* mutants, root hair nuclear movement is impaired, suggesting that a nucleo-cytoplasmic continuum SUN-WIT-Myosin XI-i may contribute to an actin-mediated nuclear movement (Tamura et al. 2013).

Altogether these data suggest that nuclear shaping may be supported by both cytoplasmic forces transmitted to the nuclear envelope and by the plamina, KAKU4 and CRWN1 may maintain nuclear morphology through interactions with the nucleocytoplasmic linker, while Nup136 may mechanically support the nuclear envelope. Among these different actors, some were shown to have a direct or indirect link with chromatin through functional and proteomic analyses. The triple mutant *sun1sun4sun5* exhibits defect in chromatin compaction and up-regulation of heterochromatin silent information such as *TSI1* (Poulet et al. 2017). NEAP3 is less tightly anchored to the inner nuclear membrane than NEAP1, and may, thus, contribute to its function in heterochromatin chromatin organisation (e.g. size and number of chromocentres, Pawar et al. 2016). CRWN4 controls higher order heterochromatin organisation and, most notably, the proper localisation of 5S RNA and centromeric repeats (Wang et al. 2013). More recently, the carrot NMCP1 protein, equivalent to CRWN proteins in *Arabidopsis*, was used as a bait to identify nuclear candidates in *Arabidopsis* using the C-terminus part of the protein involved in its nuclear periphery localisation. Four proteins were identified: the nuclear localised actin-related protein 7 (ARP7), as well as the transcription factors MYB-type transcription factor 3 (MYB3), C3HC4 RING-finger proteins (SINAT1) and BES1-INTERACTING MYC-LIKE 1 (BIM1) involved in brassinosteroid signalling (Mochizuki et al. 2017). The identification of these interacting partners may shed new light on the role of the nuclear envelope in signalling, including mechanotransduction.

Because of their localisation on both sides of the nuclear envelope, GIPs may have a unique role in this picture, notably through their association with microtubule dynamics on the one hand, and their association with centromere and chromocentres in synergy with the epigenetic regulator MGO3/TSK (Batzenschlager et al. 2017), another TSA1 partner (Takeda et al. 2004), on the other hand. Consistently, GIPs could actively contribute to heterochromatin organisation, as the *gip1gip2* mutant displays heterogeneity in chromocentres size and number (Batzenschlager et al. 2013). Interestingly, cortical microtubules change their orientation in response to mechanical cues (Hamant et al. 2008; Landrein and Hamant 2013). Although it is unclear how plant cytoplasmic microtubules behave in response to stress, this echoes the contribution of actin filaments in nuclear mechanotransduction, through the indirect interactions between actin and lamina (Enyedi and Niethammer 2016; Aureille et al. 2017). These features also do not preclude the existence of cross-talks between microtubules and actin filaments in nuclear mechanotransduction (see e.g. Sampathkumar et al. 2011 for an analysis of structural dependencies between actin filaments and microtubules in plants).

## Conclusion: avenues for future research in plants

The homologies between plant and animal potential nuclear mechanosensing pathways might echo conserved chromatin regulators and functions in both kingdoms. At the molecular level, a force in the cytoplasm will propagate to the nucleoplasm if it is not dissipated, i.e. if the LINC complex is sufficiently stiff and there is no reason to think that plants would be different from animals on that front; yet, this still needs to be formally demonstrated. Interestingly, during differentiation, or in the presence of stiffer mechanical environments, nuclear stiffness is increasing in mammals (Hampoelz and Lecuit 2011; Swift et al. 2013). This is due, in part, to the accumulation of lamins (Swift and Discher 2014) and the formation of a peripheral heterochromatin (Hampoelz and Lecuit 2011). The stiffness of the extracellular matrix has been thoroughly studied in plants, notably through decades of research on cell walls. Because cell wall stiffness can vary greatly between plant cell types or during differentiation, nuclear stiffening in cells with stiffer cell walls may also be visible in plants and help us understand how differentiation, in turn, affects gene expression. The relation between nucleus and wall stiffness has, however, not been assessed in plants so far.

Beyond the comparison between nuclear mechanosensing in plants and animals, a prospect for the future would be to unravel the actual mechanisms triggering gene expression changes. Several models involving direct mechanical perturbations on nucleus structure have been proposed: force-driven chromatin decondensation that would unmask binding sites for transcriptional regulators, force-induced chromatin detachment from the nuclear (p)lamina, moving loci away from the transcriptionally repressive nuclear periphery or force-driven conformation changes of inner nuclear envelope proteins, affecting transcriptional and chromatin regulators (Isermann and Lammerding 2013). Whether these mechanisms apply to plant nuclear mechanosensing is another exciting avenue for future research in both chromatin and mechanotransduction in plants.

Altogether, the accumulation of knowledge on the structural effectors of nuclei across kingdoms support the tensegrity concept, at least qualitatively (Ingber 2008). To demonstrate it with quantitative data will require a more thorough analysis. For instance, if microtubules, in parallel to actin, played a major role in nuclear mechanotransduction in plants, knowing that microtubules are roughly three orders of magnitude stiffer than actin filaments, at least in vitro (e.g. Gittes et al. 1993), this would inevitably affect the way forces are transduced to the nucleus. Furthermore, the added complexity of microtubule-associated proteins (and their impact on microtubule stiffness, see e.g. Portran et al. 2013) makes this endeavour both challenging and exciting.

Beyond the direct force propagation via the extracellular matrix–cytoskeleton–nuclear envelope continuum, mechanotransduction also occurs through more indirect ways. For instance, nuclear pore size may be modified in response to nuclear envelope stretching (Garcia et al. 2016). Alternatively, nuclear membrane may convert tension into biochemical signals, notably by mediating store release of Ca^2+^ at the outer nuclear membrane through mechanosensitive ion channels. For instance, the nuclear membrane protein, emerin, was recently shown to play a crucial role in nuclear structure and the production of transient nuclear Ca^2+^ peaks in animals (Shimojima et al. 2017). In that regard, nuclear pore complexes may play a similar role in calcium signalling in plants (Charpentier and Oldroyd 2013). Incidentally, a voltage-gated Ca^2+^ channel (DMI1 and CNGC15 proteins) at the nuclear envelope was recently shown to contribute to perinuclear calcium oscillation to establish plant–symbiont interactions in *Medicago* (Charpentier et al. 2016). Calcium signalling may also affect chromatin remodelling (Thuleau et al. 2012). In such a context, the role of Nup136 involved in nuclear shaping has to be explored, as well as that of TSA1 displaying a Ca^2+^ binding activity (Suzuki et al. 2005). Other indirect roles may involve the translocation of major effectors of mechanotransduction, such as β-catenin and Yap/Taz in animals (Janmey et al. 2013). The interplay between nuclear envelope mechanics and nuclear pore gating is, thus, another exciting prospect for future studies in plant nuclear mechanosensing.

Lastly, if gene expression is certainly a key aspect of mechanotransduction in development, one must recall that cells are, in principle, able to respond to mechanical cues, even without a nucleus. This was nicely shown on (enucleated) fish epidermal keratocytes, which became polar and even motile upon mechanical stimulation (Verkhovsky et al. 1999). Given the stereotypical cortical microtubule response to wall tension in plants, one may infer that this response may also not require transcriptional regulation, at least in the short term. The contribution of nuclear mechanosensing to these cortical mechanical responses, such as mechanotransduction buffering, amplification or robustness, is also likely to be a thriving field of research in the future.
